# Effect of Curing Methods on Shrinkage Development in 3D-Printed Concrete

**DOI:** 10.3390/ma13112590

**Published:** 2020-06-06

**Authors:** Karol Federowicz, Maria Kaszyńska, Adam Zieliński, Marcin Hoffmann

**Affiliations:** 1 Faculty of Civil Engineering and Architecture, West Pomeranian University of Technology in Szczecin, Al. Piastów 50, 70-311 Szczecin, Poland; maria.kaszynska@zut.edu.pl (M.K.); adam.zielinski@zut.edu.pl (A.Z.); 2 Faculty of Mechanical Engineering and Mechatronics West Pomeranian University of Technology in Szczecin, Al. Piastów 19, 70-310 Szczecin, Poland; marcin.hoffmann@zut.edu.pl

**Keywords:** 3D Concrete Printing, curing conditions, shrinkage, digital construction, additive manufacturing

## Abstract

Technological developments in construction have led to an increase in the use of 3D modelling using CAD environments. The popularity of this approach has increased in tandem with developments in industry branches which use 3D printers to print concrete based printing materials in construction, as these allow freedom in shaping the dimensions of supporting elements. One of the biggest challenges for researchers working on this highly innovative technology is that of cement material shrinkage. This article presents the findings of research on an original method of measuring deformations caused by shrinkage in 3D-printed concrete elements. It also discusses the results of tests on base mixes, as well as comparisons between the influence of internal and external curing methods on the development of deformations and their final outcomes. Furthermore, the article discusses differences between deformations formed after seven days of hardening without curing, with those which occur when two common, traditional concrete curing methods are used: foil insulation and shrinkage reducing admixtures. In addition, the article examines the effects of internal curing on the 1, 7, 14, 21 and 28 day mechanical properties of concrete, in accordance with EN 196-1 and EN 12390-2. Studies have shown that the optimal amount of shrinkage reducing admixtures is 4% (in relation to the mass of cement), resulting in a reduction in total shrinkage of 23%. The use of a shrinkage reducing admixture in 3D-printed concrete does not affect their strength after 28 days, but slows the strength development during the first 7 days.

## 1. Introduction

### 1.1. Modern Technology in Construction

The last few years have witnessed considerable advancements in the construction field, which has widely begun to adopt automated and computerised industrial processes [1]. CAD environments are being used in both conceptualising and creating architectural projects, while constructors are utilising advanced software packages for their numerical calculations. Developments in these two fields are occurring in tandem with those in Building Information Modelling (BIM), which allows automatic data flows and creates standards for data exchange. What follows from the application of such technologies is automation at the execution stage of a construction, this being observable through the developments occurring in the production of prefabricated construction elements. One of the innovations which has emerged in recent years, is that of using concrete mixes in additive manufacturing (3DCP). It is also possible to use many other materials in additive manufacturing, such as steel, clay, geopolymers, glass or synthetic materials [2,3,4,5,6]. Despite the fact that methods of 3D printing with each of these materials are at varying levels of development (Technology Readiness Level), they all allow for optimisation and changes in the methods used in construction [2,3,4,5,6].

3D printing utilising concrete mixes is currently one of the most rapidly developing fields in construction; the best evidence of this is the number of new research projects and private endeavours focusing on this technology worldwide [7,8,9,10].

However, despite the intensive research being carried out at many research institutes, the introduction of this technology on an industrial scale is still associated with numerous unsolved material questions and construction-related problems. Much research has focused on the influence of the level of adhesion between layers on the durability of the entire element, as well as on the mechanical behaviour of mixes during the 3D printing process. [11]. The difference between 3DCP elements and traditional concrete elements is strength, depending on the direction of load application. Traditional cubic concrete samples show constant mechanical properties in all directions, while printed elements, due to their layered structure, show strong orthotropic properties. This is due to the very low adhesion of layers and low interlayer bind, a phenomenon confirmed in recent studies [12]. The mixes used in 3D printing achieve high levels of mechanical performance in short periods of time and their compacted structures, resulting from their fine-grained compositions, allow them to support their own weight as well as that of successive layers, with minimal deformations [1,13]. These mixes are characterised by a high proportion of cement and mineral additives such as fly ash, silica fume or limestone powder [14,15]. Only fine aggregates with a maximum size of 2 mm are used, because of the small cross-section of the printing samples and the necessity of pushing the mix through a pump.

### 1.2. Background and Motivation

Mix composition and the immediate onset of the phenomenon of drying bring about a number of problems related to the dynamic development of shrinkage deformations in the printed material. High binder contents, the lack of internal limitations from coarse aggregates and the erection of elements without formwork, exposes printed materials to a lot of quickly occurring shrinkage caused by drying and self-drying [16]. The drying of a concrete mix in the first hours after mixing with water is made possible by the ability of moisture to quickly permeate fresh concrete [17]. The onset of deformations in the material during this period is referred to as plastic shrinkage [18]. It results from a concrete mix losing moisture as a result of its movement towards equilibrium with the moisture of the surroundings [19]. The rate and extent of plastic shrinkage depend on numerous factors, including the size of the area susceptible to drying, the moisture and temperature of the ambient conditions, as well as air flow [20]. Furthermore, the accelerated hardening of a mix with a low volume of water also has an effect on autogenous shrinkage. With its mechanical properties only in their beginning phases, fresh concrete has a low ability to resist expansion, thereby making stress transfer impossible, which leads to fractures and cracks in cementitious elements. In turn, these can lead to an erosion of durability and weight-bearing capacity, thereby generating high repair costs [20]. In most cases, the parameters mentioned above cannot be controlled during printing and it is thus vital that early age onset shrinkage is minimised. Both active and passive methods, based on curing as well as on techniques for strengthening the structure of a composite, are used to keep deformations in traditional concretes to a minimum. Passive methods are based on having the appropriate mix design, using anti-shrink fibres such as those made of glass, polypropylene, polyethylene, basalt or steel [21,22,23], or incorporating internal curing methods, such as adding shrinkage-reducing agents (SRA) [24,25,26] or using fine aggregates soaked in water [27]. Active methods, on the other hand, are characterised by the actions taken to prevent the evaporation of water from the surface of the material. Such methods include spraying the surface of the concrete with water, covering it with anti-evaporation agents (membrane waterproofing) or foil, as well as shielding the concrete from direct exposure to sunlight and wind [28].

This article presents the results of tests and analyses related to the influence of both active and passive traditional concrete curing methods on the development of 3D-printed concrete shrinkage as well as on its properties regrading durability. Two types of curing were adopted: external curing based on isolating samples with foil, as well as internal curing using anti-shrinkage admixture (SRA) in the amounts of 2% and 4% per weight of cement.

## 2. Materials and Methods

### 2.1. Materials

The concrete mixes under investigation were prepared with CEM I 52,5R Portland cement (European Standard EN 197-1:2011 [29]), fly ash, silica fume, superplasticizer and fine aggregate (0–2 mm). The composition and notification of the investigated mixtures are presented in Table 1. The w/c (water to cement) and w/b (water to binder) ratios were assumed to be 0.34 and 0.28, respectively, whereas c/a (cement to aggregate) ratios was 0.47 and b/a (binder to aggregate) was equal to 0.67. All the ratios were calculated in accordance to the mass of products. The base mix design adopted in this study was developed by the authors, as the result of numerous—mainly unpublished—tests and experiments related to 3D printing technology [30,31,32].

Three 1350-mm-long samples of concrete were printed from each mix for shrinkage tests. Furthermore, typically casted 40 mm × 40 mm × 160 mm prism specimens were also made for the purposes of compressive strength testing in accordance with EN 196-1 [33] and flexural strength testing according to EN 12390-2 [34], after 1, 7, 14, 21 and 28 days. The consistency of the concrete mixes was determined using the slump flow test, according to EN 1015-3. [35]

Both external and internal curing were utilised in the experiment: the former method consisted of isolating the printed specimens with foil, while the latter method involved adding shrinkage reducing agents (SRA) in the amounts of 2% and 4% per mass of cement. Water volume was reduced when hydrated SRA was added to the mixes, thus maintaining both a constant w/c coefficient and consistency.

### 2.2. 3D Printer

**Figure 1:**
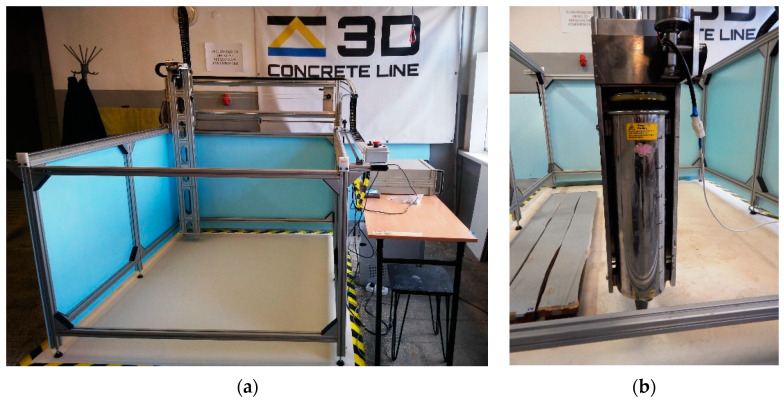
3D concrete printer: (**a**) work space; (**b**) extruder module.

A custom 3D printer, with the kinematics of a cartesian coordinate robot [30], was used to print the specimens used for the shrinkage testing. Its working area in the horizontal plane is around 145 cm × 145 cm, whilst in the vertical plane, it is 90 cm. As presented in Figure 1, two printing modules can be connected to it: a pump for constant printing and an extruder for smaller print jobs of up to seven litres. The tests reported in this paper were undertaken using the extruder module. The printer was steered using a G-code. The nozzle used had a circular cross section of 20 mm. The print head operated at a constant speed of F = 3000 mm/min, with a constant extrusion rate of 1.58 L/min.

**Figure 2:**
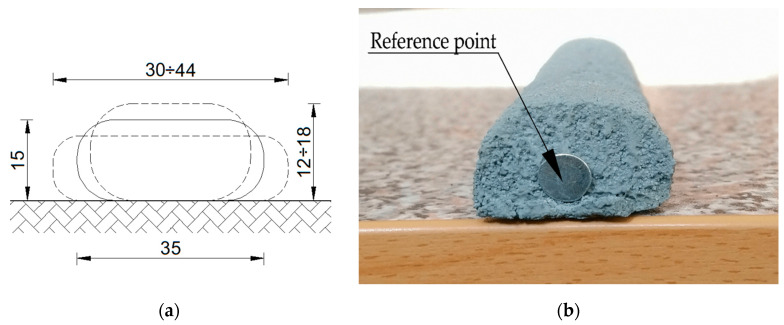
Specimen cross-sections: (**a**) diagram; (**b**) actual printed matter containing the reference point.

The length of the printed sample—limited by the printer’s maximum working area length—was 1350 mm, while the extruded layer was approximately 35 mm in width and 15 mm in height (Figure 2). Only one layer was printed and its deformations examined, in order to eliminate the effects of subsequent layers. The printed layers were placed on a sheet of foil, so as to eliminate restrained shrinkage of the mix, which may have occurred as a result of adhesion or friction between the contact surface and the pedestal. As a result, there was no possibility of the specimens cracking or fracturing because of tensile stress arising from mix shrinkage.

### 2.3. Shrinkage Measurements

**Figure 3:**
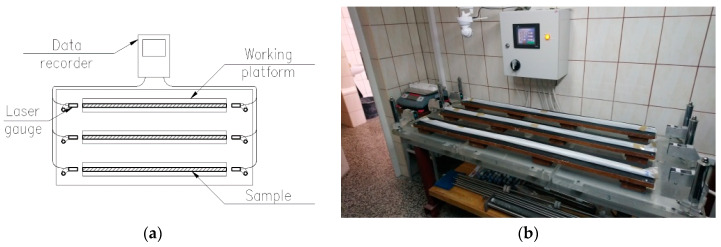
The testing site: (**a**) diagram; (**b**) actual site.

A novelty of the presented approach is to test the total shrinkage of the mixtures used for 3D printing starting just after the addition of water to mixture. The proposed method enables the measurement of volumetric changes taking place in the initial stages of material maturation—especially during the first 24 h. There is a lack of research that analyzes this phenomenon. Standardized measurement methods based on Graf-Kaufman, Amsler tests or similar tests proposed in ASTM C157 [36] make it impossible to measure the shrinkage of 3D-printed concretes, as the measurement takes place after 24 h. In those traditional methods, samples mature for 24 h in moulds, which protects them from drying shrinkage. The shape of prisms and their smooth surface—due to vibrating and compacting—do not reflect the geometry of layers of printed concrete. Because of this, the authors proposed a method where laser measurement sensors were installed at the testing site. These facilitated contactless measurement of deformations in the printed product and the elimination of potential, externally caused deformations. As shown in Figure 3, the use of the sensors made it possible to measure the deformations three specimens at a time. As shown in Figure 2b, the end of each sample was fitted with steel reference points which had flat and smooth frontal surfaces. The reference point had the shape of a flat-headed pin with a diameter of 3 mm and a length of 30 mm, with a flat surface on one side with a diameter of 8 mm and a weight of 1.55 g. The sensors worked by picking up the light reflected from the front of the reference point and measuring its movement over time. The distance between the sensors and the reference point was between 40 and 60 mm, with the initial distance at the onset of printing set at 50 mm. Measurements were made every 60 s to an accuracy of 2 µm. The first reading was taken within ten minutes of sample extrusion and within thirty minutes of water being added to the mix. The entire testing site was situated in an environmental chamber, which guaranteed a constant temperature (T = 20 ± 3 °C) and humidity (RH = 50 ± 3%) while preventing air flow.

Throughout the entire testing procedure, the specimens produced for the purposes of shrinkage testing were placed on a layer of foil, which was situated on the working platform. At the end of the printing procedure, excessive material was removed from the extruded samples so as to adhere to the desired sample length of 1350 mm, after which the reference points were installed. Next, the specimens which were to harden underneath foil were hermetically sealed in it, with care taken so as not to cause any deformations. All the specimens were immediately transferred to the environmental chamber, at which point—after installation of the reference points—the process of deformation measurement began.

### 2.4. Compressive and Flexural Strength Measurements

To determine the influence of the SRA admixture on the mechanical properties of the concrete, typically casted 40 mm × 40 mm × 160 mm specimens were produced, according to EN 196-1. For each type of concrete, three specimens were produced for each testing time. After 1, 7, 14, 21 and 28 days, the specimens were tested for their flexural and compressive strengths. A standard testing machine, in accordance with EN 196-1, was used for measurement. The specimens produced for strength testing were formed and stored in accordance with EN 12390-2.

## 3. Results

### 3.1. Shrinkage Test Results

**Scheme 1:**
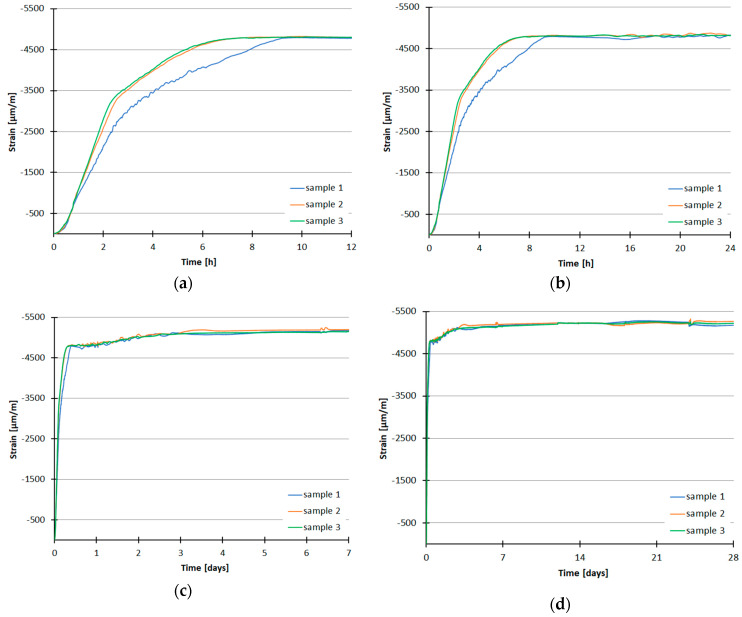
C580/SF83/FA166 concrete shrinkage after: (**a**) 12 h; (**b**) 1 day; (**c**) 7 days; (**d**) 28 days.

The measurement of shrinkage deformations in the base mix, which did not contain any SRA, was carried out for 28 days. This involved measurement of the total shrinkage, which was a sum of both autogenous shrinkage and drying shrinkage [17,20,21,22]. The average shrinkage value measured on the 28th day was −5269 µm/m, with the standard deviation not exceeding 327 µm/m. Scheme 1a–d, respectively, shows the progression of shrinkage for each of the three specimens, after 12 h, 24 h, 7 days and 28 days.

**Scheme 2:**
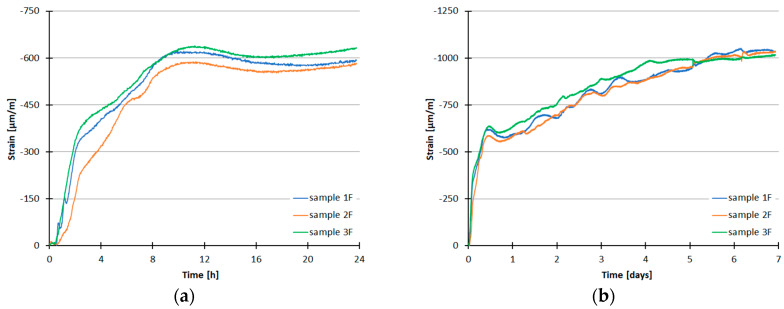
C580/SF83/FA166 concrete (isolated in foil) shrinkage after: (**a**) 1 day; (**b**) 7 days.

The cured specimens were measured for seven days. Further measurements were not carried out because deformations ceased to appear after the first week of maturation. The maximum average deformations, in the specimens where drying was minimised via foil isolation, measured −1031 µm/m. Scheme 2a,b presents the progression of deformations caused by shrinkage of the mix in the first 24 h and after seven days, with the standard deviation not exceeding 78 µm/m.

**Scheme 3:**
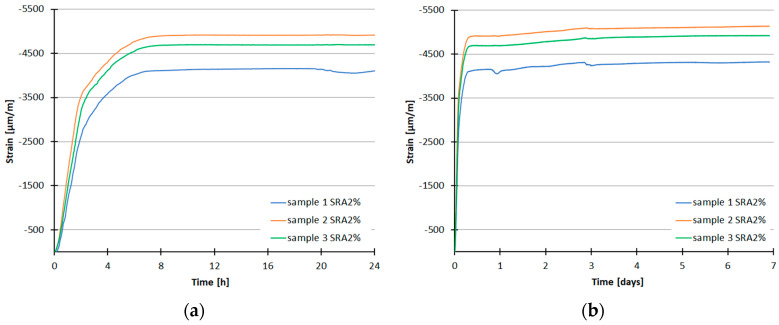
C580/SF83/FA166/SRA2 concrete shrinkage after: (**a**) 1 day; (**b**) 7 days.

**Scheme 4:**
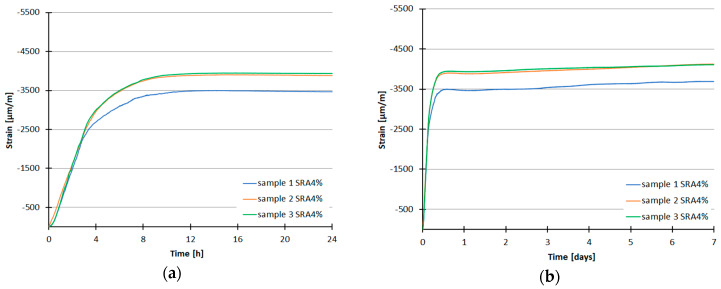
C580/SF83/FA166/SRA4 concrete shrinkage after: (**a**) 1 day; (**b**) 7 days.

The results of the measurements of shrinkage deformations in mixes containing a 2% addition of SRA have been presented in Scheme 3, with Scheme 4 showing the results for mixes containing 4% of SRA. The average deformation sizes after seven days were −4797 and −3976 µm/m, respectively. The standard deviation varied according to the age of the cement, with values between 5 and 383 µm/m for the mixes with 2% of SRA and 3 and 221 µm/m for the mixes with 4% of SRA.

### 3.2. Compressive and Flexural Strength Tests

Compressive and flexural strength tests were carried out on normalised samples for all of the mixes, in accordance with EN 196-1, after 1, 7, 14, 21 and 28 days. The samples were kept in an environmental chamber at a temperature of 20 ± 2 °C and a relative humidity of RH = 65 ± 5%. Table 2 shows the results of compressive strength tests carried out on the base mix (C580/SF83/FA166), mixes containing 2% shrinkage-reducing admixture (C580/SF83/FA166/SRA2) and mixes containing 4% shrinkage-reducing admixture (C580/SF83/FA166/SRA4), with Table 3 showing the results of flexural strength tests for these mixes.

## 4. Discussion

**Scheme 5:**
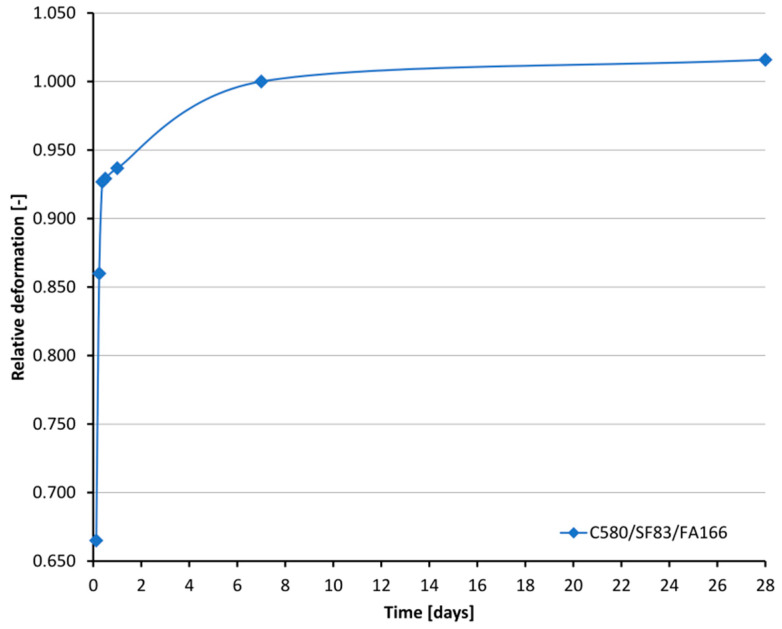
Relative increase in shrinkage for mix C580/SF83/FA166.

According to the obtained results, it can be seen that the increase in deformations in specimens subject to drying stabilised after three days. Table 4 presents the percentage increases in deformations, as well as their dimensions, after seven days. As can be seen in Scheme 5, measurements taken over a period of 28 days for mix C580/SF83/FA166, show that a small increase in shrinkage occurred between day seven and day 28. In this instance, it is presumably autogenous shrinkage, rather than drying shrinkage, which was dominant. Taking the seven day shrinkage result as a point of reference, increase in shrinkage over the next 21 days was only 1.6%.

For all samples subject to drying, the largest increase in deformation occurred in the first three hours after printing. This amounted to around 66% of all the deformations in mix C580/SF83/FA166, and 77% and 61% of deformations in the mixes with 2% and 4% of SRA, respectively. The mixes with 2% SRA showed more shrinkage, and at a higher rate of increase, than those with 4% SRA. A decrease in shrinkage-reducing admixture from 4% to 2% resulted in an increase in the scale and rate of shrinkage in the first six hours. Adding SRA in the amount of 4% per mass of cement reduced both the total amount of deformations, as well as their rate of increase, in the first nine hours. In the case of the samples isolated by foil, the growth of deformations was slower and more uniform, which is characteristic of autogenous deformations over time [37]. The samples subjected to drying achieved approximately 95% of their total deformations in the first 24 h. The characteristic of the deformations necessitates measurement immediately from the moment of samples being printed.

**Scheme 6:**
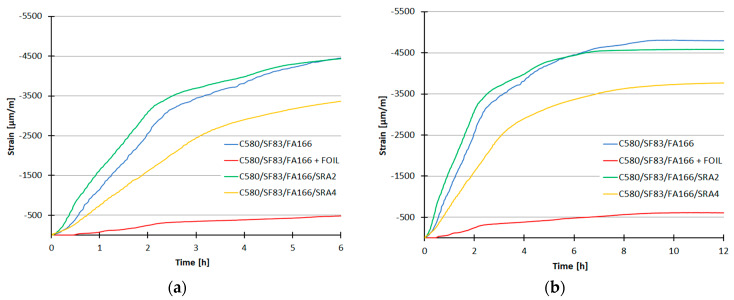
Average deformation over time: (**a**) 6 h; (**b**) 12 h.

**Scheme 7:**
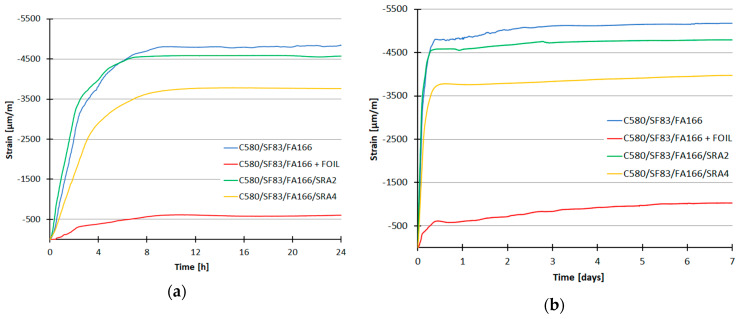
Average deformation over time: (**a**) 24 h; (**b**) 7 days.

A comparison of the shrinkage deformations in all the concrete samples tested, measured at 6 h, 12 h, 24 h and 7 days, can be found in Scheme 6 and Scheme 7. The samples isolated with foil showed a significant difference in the progression of shrinkage. The application of external curing stabilised deformation growth equally at both the six- and twelve-hour points, with the progression being almost linear and with only a small gradient (i.e., a weak development of deformations, of a primarily autogenous nature). Over a period of seven days, autogenous shrinkage caused a slow growth in deformations in the externally cured samples.

The manner in which the deformations formed was very similar across all of the samples subjected to drying, irrespective of the internal curing method applied. Both the base mix and the mix with 2% SRA had similar gradients after the first six hours, indicating similar deformation growth rates. A shifting of the slope in the graph for cement C580/SF83/FA166/SRA2 towards the vertical axis, was a result of the difference in the time which lapsed between sample production and the beginning of deformation measurement between these samples and the base samples. The gradient was much smaller for the concrete with an SRA content of 4%, with the progression of concrete deformation being much smoother. A stabilisation in the increase in deformations occurred for all mixes in the next six hours. From this point on, shrinkage in the base concrete was higher than in the concretes containing SRA.

The deformation growth rate in the base concrete, as well as in that with added SRA, was clearly suppressed in the first 24 h of measurement. This is a point in time at which the hardening of a mixture has finished and when the cement matrix is so compacted that there is no more moisture loss. A very small increase in deformations, resulting from autogenous shrinkage in the material, can be observed between the first and seventh days of measurement. The gradients of all of the graphs are convergent, which confirms that the cause of deformation was autogenous shrinkage (to a large extent dependent on the amount of binder in the mix [38,39,40]).

In the seven day period analysed, in comparison to the base concrete, the 2% and 4% additions of SRA caused 7% and 23% reductions in shrinkage, respectively. The most significant reduction in shrinkage occurred in the concrete cured externally with foil covering; this had only 20% of the deformations of the base mix. Accordingly, external curing was the most effective method for limiting shrinkage in the concrete under analysis, with no fractures or cracks observed for any of the samples measured.

The use of shrinkage-reducing agents did not significantly affect the compressive strengths of the mixes analysed in this study, with the differences after 28 days being around 4%. However, it should be noted that an increasing SRA dosage resulted in a progressively slower increase in strength, in the first seven days. This relationship was not observed in the case of bending tensile strength, but both mixes with added SRA achieved higher strengths after 28 days, which might have been a result of the minimisation of microcracks in the cement matrix and its more compacted structure.

## 5. Conclusions

### 5.1. Findings Summary

On the basis of the tests undertaken in this study, it is clear that measurement must start immediately after sample printing, as a result of the way in which shrinkage progresses. In many articles about high-performance concrete (HPC), which has similar mechanical properties to 3D-printed concrete, authors focus on autogenous shrinkage. For concrete with a low water-to-cement ratio (w/c = 0.2–0.4) deformation caused by autogenous shrinkage varies from 330 to 850 µm/m [37,41,42,43]. According to the literature, total shrinkage of HPC with a w/c ratio between 0.25 and 0.36 measured in accordance with ASTM C157 rarely exceeds 800 µm/m [44,45,46,47]. In all this research, concretes were made using coarse aggregate which, in general, limits the shrinkage. Nevertheless those results were obtained for long-term measurements—between 28 and 365 days long. Some research focuses on the shrinkage of mortars (with compressive strength exceeding 50 MPa), which are more similar to presented mixes in terms of aggregate composition—lack of coarse aggregate. Most of them are based on the Amsler and ASTM C157 tests. The long-term total shrinkage of those mortars is approximately 1200 µm/m [48,49,50]. For printed concrete, many researchers emphasized the problem of total shrinkage and recommend investigating it, but do not present sufficient results. Most of them are based on measurement of the material that can be used in 3D printing, but samples for the shrinkage test are prepared in a traditional way, which lead to results c.a. 1500 µm/m after 28 days [51]. Such shrinkage value is related to omitting the influence of drying of the fresh mix and autogenous shrinkage in the first day, which is important for mixtures with a large amount of binder. In the presented research, the authors measured total shrinkage that was about three times higher than described in previous articles. This phenomenon strongly indicates that the idea proposed by the authors of recording total shrinkage from the very beginning was right. The measurement equipment used and the methodology applied are ideal for the analysis of cementitious materials to be used in 3D printing. The method proposed in this paper makes it possible to quickly examine the influence of the hardening conditions in place and the curing methods chosen on the shrinkage of printed elements. On the basis of the measurements taken and the analyses performed, it is recommended that measurement should be carried out for seven days, after which the deformation stabilises. When producing elements with the use of 3DCP, the highest possible amount of anti-shrinkage admixture should be used. If 2% per mass of cement of admixture is used, according to manufacturer recommendations, shrinkage will only be reduced by 7%. However, it is possible to achieve a reduction in shrinkage of around 23%, if 4% of admixture is used instead. The most effective curing method was found to have been external curing, which reduced shrinkage from 5178 to 1031 µm/m. However, this form of curing limits the printing of subsequent layers during continuous printing, thus making it inappropriate for industrial purposes. The use of internal curing can simplify the process of 3D-printing construction, by eliminating the need to cover elements with foil. Furthermore, the SRA dosage can be more effectively controlled when it is applied during the production of the mix, thus increasing its efficacy. Consequently, a two pronged curing method, utilising both internal and external methods, would be most effective. For internal curing, the method proposed would involve the use of an SRA admixture at a volume much higher than in the case of traditional concrete. This should be supplemented with a non-invasive method of external curing: the use of membrane-forming preparations. Insofar as control of the ambient environmental conditions is possible, the temperature should be kept constant and below 22 °C, with a relative humidity above 70%.

While anti-shrinkage admixtures do not have a significant effect on compressive strength after 28 days, they nonetheless suppresses the rate of its increase. This should be kept in mind when producing elements via 3DCP, as these are usually placed under load-bearing soon after their erection.

### 5.2. Future Work

In analysing the results presented in this paper, as well as the most recent literature in the field of 3D concrete printing, it would be prudent to consider future possible research avenues regarding deformations in production materials, as well as to analyse methods of predicting shrinkage in 3DCP [52,53]. The research shows the influence of the types of curing method on the shrinkage of 3D-printed concrete. The measurement method proposed in the article allows to verify the effectiveness of the applied method in reducing material shrinkage. In order to adapt laboratory tests to industrial applications, further research will investigate five-layer samples and measure the deformation of the bottom, middle and top layers. Preliminary analysis shows that the shrinkage of individual layers is not the same. The highest shrinkage value is observed for the upper layer and the lowest for the bottom layer. Further work will also focus on comparing interactions between anti-shrinkage admixtures based on different chemical compositions, examining the effects of using fibres, as well as on external curing utilising surfactants. Efforts should also be made to systematise methods for analysing 3DCP mix shrinkage, so as to be able to easily and unequivocally compare mixes developed in different parts of the world.

## 6. Patents

No. PL231299: Method for measuring the self-generated shrinkage of poured out composite materials and the workstand for measuring the self-generated shrinkage of poured out composite materials

## Figures and Tables

**Table 1:** Composition and notification of concrete mixes.

Concrete	Cement 52,5R [kg/m^3^]	SCM [kg/m^3^]		Water [kg/m^3^]	SP [kg/m^3^]	SRA [kg/m^3^]	Aggregate [kg/m^3^]
		Fly Ash	Silica Fume				
C580/SF83/FA166	580	166	83	200	18	-	1234
C580/SF83/FA166/SRA2	580	166	83	189	18	11.6	1234
C580/SF83/FA166/SRA4	580	166	83	177	18	23.2	1234

**Table 2:** Compressive strength.

Mixes	Compressive Strength [MPa]				
	1 Day	7 Days	14 Days	21 Days	28 Days
C580/SF83/FA166	35.14	71.81	79.47	81.36	84.61
*Standard Dev.*	*3.96*	*8.15*	*12.27*	*9.46*	*10.31*
*CoV*	*0.11*	*0.11*	*0.15*	*0.12*	*0.12*
C580/SF83/FA166/SRA2	27.70	68.02	77.29	84.06	88.23
*Standard Dev.*	*2.04*	*1.89*	*1.60*	*3.68*	*3.72*
*CoV*	*0.07*	*0.03*	*0.02*	*0.04*	*0.04*
C580/SF83/FA166/SRA4	23.59	59.64	72.60	79.06	84.90
*Standard Dev.*	*0.91*	*3.09*	*4.57*	*5.07*	*2.61*
*CoV*	*0.04*	*0.05*	*0.06*	*0.06*	*0.03*

**Table 3:** Flexural strength.

Mixes	Flexural Strength [MPa]				
	1 Day	7 Days	14 Days	21 Days	28 Days
C580/SF83/FA166	6.99	11.57	13.59	14.70	14.95
*Standard Dev.*	*1.18*	*1.04*	*1.73*	*1.40*	*1.41*
*CoV*	*0.17*	*0.09*	*0.13*	*0.10*	*0.09*
C580/SF83/FA166/SRA2	7.07	11.63	12.36	15.80	18.39
*Standard Dev.*	*0.15*	*0.21*	*0.27*	*0.44*	*0.74*
*CoV*	*0.02*	*0.02*	*0.02*	*0.03*	*0.04*
C580/SF83/FA166/SRA4	5.42	9.69	14.27	15.16	17.36
*Standard Dev.*	*0.37*	*0.78*	*0.06*	*0.21*	*0.36*
*CoV*	*0.07*	*0.08*	*0.004*	*0.01*	*0.02*

**Table 4:** Concrete shrinkage over time.

Time [h]	Shrinkage Strain							
	C580/SF83/FA166		C580/SF83/FA166 + FOIL		C580/SF83/FA166/SRA2		C580/SF83/FA166/SRA4	
	[μmm]	[%]	[μmm]	[%]	[μmm]	[%]	[μmm]	[%]
3	−3443	66.5	−347	33.6	−3695	77.1	−2444	61.5
6	−4452	86.0	−484	47.0	−4437	92.5	−3367	84.7
9	−4798	92.7	−599	58.0	−4576	95.4	−3693	92.9
12	−4811	92.9	−615	59.6	−4586	95.7	−3769	94.8
24	−4850	93.7	−612	59.3	−4589	95.7	−3784	95.2
7 days	−5178	100.0	−1031	100.0	−4795	100.0	−3976	100.0
28 days	−5260	101.6	-	-	-	-	-	-
